# From Granulomas to Malignancy: Validating the Sydney System in a Tuberculosis-Endemic Landscape

**DOI:** 10.7759/cureus.111266

**Published:** 2026-06-21

**Authors:** Joyeeta Mandal, Sona Pathak, Arpana S Tirkey, Susruta Sen, Minakshi Karmakar, Nirmalya Banerjee, Sudip Roy, Nivedita Bhattacharjee

**Affiliations:** 1 Pathology, C K Birla Hospital, The Calcutta Medical Research Institute, Kolkata, IND; 2 Pathology, Mahatma Gandhi Memorial (MGM) Medical College and Hospital, Jamshedpur, IND; 3 Laboratory Medicine, C K Birla Hospital, The Calcutta Medical Research Institute, Kolkata, IND; 4 Histopathology, C K Birla Hospital, The Calcutta Medical Research Institute, Kolkata, IND; 5 Microbiology, C K Birla Hospital, The Calcutta Medical Research Institute, Kolkata, IND

**Keywords:** cytopathology, diagnostic accuracy, granulomatous lymphadenitis, lymph node fnac, risk of malignancy, sydney system for reporting lymph node cytopathology, tuberculosis

## Abstract

Background: The Sydney System for reporting lymph node cytopathology standardizes lymph node fine-needle aspiration cytology (FNAC) reporting and improves risk-based clinical stratification. Validation data from tuberculosis (TB)-endemic regions remain limited. Materials and methods: This retrospective observational multicentric study analyzed 427 consecutive lymph node FNAC cases from two tertiary care centers in Eastern India during January-December 2025. Three independent pathologists, reviewing slides at separate time points, categorized aspirates according to the Sydney System (L1-L5), blinded to clinical details and to each other’s assignments. Histopathology and Ziehl-Neelsen staining served as reference standards. Diagnostic accuracy parameters with 95% confidence intervals (Wilson score method), receiver operating characteristic (ROC) analysis, category-wise risk of malignancy (ROM), TB correlation with a complete 2 × 2 matrix, and interobserver agreement (Cohen’s kappa) were assessed. Results: Cervical lymph nodes constituted 357 (83.6%) cases. Reactive, granulomatous, and necrotizing lymphadenitis accounted for the majority of diagnoses; malignant lesions accounted for 39 cases (9.1%). The L2 (benign/reactive) category constituted the overwhelming majority of cases, with a mean of 384 (89.9%) across pathologists out of 427 cases. Per-pathologist sensitivity ranged from 79.5% to 84.6%; specificity was 99.7% across all three observers; positive predictive value was 96.9-97.1%; negative predictive value was 98.0-98.5%; and overall accuracy was 97.9-98.4%. ROC analysis demonstrated excellent discriminatory performance (AUC range: 0.935-0.974). ROM increased progressively from L2 to L5. Interobserver agreement was substantial to almost perfect (κ = 0.79-0.87). Among L2 cases, the sensitivity for TB capture was 98.6%; however, the PPV of L2 for TB was only 19.2%, and specificity was 13.0%, with 307 (80.8%) cases of granulomatous L2 findings attributable to non-TB aetiologies. Conclusions: The Sydney System demonstrates excellent diagnostic accuracy, reproducibility, and clinically meaningful ROM stratification in a TB-endemic setting. Granulomatous cytomorphology alone is insufficient for TB diagnosis; microbiological confirmation is mandatory before assigning an L2 classification to TB.

## Introduction

Peripheral lymphadenopathy is a common clinical presentation with a broad differential diagnosis ranging from reactive and infectious conditions to primary lymphoid malignancies and metastatic carcinoma. In tuberculosis (TB)-endemic countries such as India, tuberculous lymphadenitis remains one of the leading causes of peripheral lymphadenopathy, particularly in the cervical region. Accurate distinction between benign inflammatory lesions and malignancy is therefore essential for appropriate patient management [1].

Fine-needle aspiration cytology (FNAC) is widely used as the initial diagnostic modality for lymph node evaluation because it is minimally invasive, rapid, cost-effective, and suitable for resource-limited settings. [2] In addition to cytomorphological assessment, FNAC material can be utilized for ancillary investigations such as Ziehl-Neelsen (ZN) staining, microbiological culture, immunocytochemistry, flow cytometry, and molecular testing. Previous studies have demonstrated the high diagnostic utility of lymph node FNAC in both benign and malignant conditions [2,3].

Despite its widespread use, lymph node cytology historically lacked a universally standardized reporting system, leading to interobserver variability and inconsistent communication between pathologists and clinicians. To address this, Field et al. introduced the Sydney System for reporting lymph node cytopathology in 2020 as a standardized five-tier classification comprising L1 (inadequate/nondiagnostic), L2 (benign), L3 (atypical cells of undetermined significance/atypical lymphoid cells of uncertain significance), L4 (suspicious for malignancy), and L5 (malignant) [4]. Each category carries an estimated risk of malignancy (ROM) and corresponding clinical recommendations, improving diagnostic reproducibility and patient triage. The World Health Organization subsequently adopted and extended this framework in its 2025 reporting system for lymph node, spleen, and thymus cytopathology, providing further refinement of diagnostic criteria and management guidance [5,6].

Several validation studies from Indian institutions have supported the clinical utility of the Sydney System, demonstrating a progressive increase in ROM across categories, high specificity for malignancy prediction, favorable diagnostic accuracy in head and neck lymphadenopathy, and substantial interobserver agreement among pathologists [7-10].

However, while these studies have established the Sydney System's accuracy for malignancy detection and its category-wise risk stratification, none has systematically quantified the diagnostic performance of the L2 category specifically for TB using a contingency analysis inclusive of specificity and PPV. Given that granulomatous and reactive lesions dominate L2 in TB-endemic settings, this represents a clinically significant gap: a high sensitivity of L2 for TB capture can mask poor specificity, leading to overdiagnosis and inappropriate empirical anti-tubercular therapy. The present study addresses this gap directly, providing what is, to our knowledge, among the first complete TB-specific diagnostic accuracy analyses nested within a Sydney System validation framework in an Eastern Indian, TB-endemic, multicentric cohort.

India continues to bear the highest global burden of TB [11]. Tuberculous lymphadenitis represents a major diagnostic challenge because granulomatous cytomorphology is not pathognomonic for TB and may also occur in fungal infections, sarcoidosis, Kikuchi disease, and certain malignancies [12,13]. Microbiological confirmation using ZN staining, culture, or molecular methods therefore remains essential for definitive diagnosis [13].

The present multicentric study was undertaken to validate the Sydney System for reporting lymph node FNAC at two tertiary care centers in Eastern India. The objectives were to assess the diagnostic accuracy of FNAC in detecting malignancy, determine the ROM within individual diagnostic categories, evaluate the association between granulomatous lymphadenitis and microbiologically confirmed TB, and analyze interobserver agreement among independent pathologists.

## Materials and methods

Study design and setting

This retrospective observational study was conducted at a tertiary-care semi-urban teaching hospital and a tertiary-care urban multispecialty hospital, both serving the Eastern Indian region. FNAC slides and confirmatory data from cases presenting during a 12-month period (January-December 2025) were retrieved and analyzed retrospectively. Ethics committee approval was obtained from the Institutional Ethics Committee of the Calcutta Medical Research Institute (approval number: CMRI/IEC/Patho/05-22/1566) and the MGM Medical College and Hospital (approval number: IEC/PATHO/299/2025). Patient data were anonymized in accordance with the ICMR National Ethical Guidelines for Biomedical and Health Research Involving Human Participants.

Study population

A total of 427 patients with clinically suspected lymphadenopathy who underwent FNAC at the two institutions were enrolled. Inclusion required: (a) availability of FNAC slides and (b) histopathological confirmation for all cases where malignancy was suspected or diagnosed (L3-L5 categories and any case with clinical suspicion), plus ZN staining for all granulomatous or necrotizing aspirates. For benign L1-L2 cases without clinical suspicion of malignancy in which histopathological biopsy was not clinically indicated, non-malignant status was inferred from a composite of cytomorphology, ZN staining results, clinical follow-up, and, where available, microbiological culture. This approach is consistent with published validation methodologies for the Sydney System, in which composite noninvasive criteria have been accepted as a valid reference standard for benign categories when histopathological biopsy is not clinically indicated; the exact number of cases confirmed by this method is reported in the Results. Cases with insufficient material for assessment were retained as L1 and included in the analysis. Cases with no confirmatory data of any kind were excluded.

FNAC procedure and cytological assessment

FNAC was performed using 22-25G needles under standard aseptic conditions. Smears were air-dried and stained with Leishman stain and May-Grunwald-Giemsa stain; selected smears were alcohol-fixed and stained with Papanicolaou stain. ZN staining was performed on all aspirates demonstrating granulomatous or necrotizing inflammation. Three independent pathologists, blinded to clinical details, final diagnoses, and to each other’s classifications, categorized FNAC findings according to the Sydney System (L1-L5). Each pathologist reviewed all slides at different time points to prevent recall bias. Discordant cases (defined as any disagreement among the three observers) were resolved by consensus review at a multi-headed microscope session; consensus classifications were used solely for final diagnostic purposes, and kappa analysis was performed on the original pre-consensus classifications to preserve independence.

Final diagnosis and reference standard

The reference standard for malignancy was histopathological examination of core-needle, incisional, or excisional biopsy specimens. For cases in which a biopsy was not performed (predominantly L1-L2 benign cases without clinical suspicion), non-malignant status was established using the composite criteria described in the Study Population section; the number of such cases is reported in the Results. TB diagnosis required microbiological confirmation by ZN staining positivity. One case with ZN positivity was classified as lepromatous leprosy on histopathology (*Mycobacterium leprae* is weakly acid-fast and ZN-stain-positive); this case was placed in the non-TB benign category. For statistical purposes, TB was classified as a non-malignant diagnosis.

Statistical analysis

Statistical analyses were performed using SPSS Statistics version 25 (IBM Corp. Released 2017. IBM SPSS Statistics for Windows, Version 25.0. Armonk, NY: IBM Corp.). Descriptive statistics were presented as distributions of patient demographics, FNAC sites, cytopathological diagnoses, and Sydney System categories per pathologist.

Diagnostic accuracy for malignancy in terms of sensitivity, specificity, positive predictive value (PPV), negative predictive value (NPV), and overall accuracy was calculated for each pathologist by comparing Sydney System categories (L4/L5 = FNAC-positive; L1-L3 = FNAC-negative) against confirmed malignancy status (histopathology). This dichotomization was consistent with prior Sydney System validation studies, where L4-L5 constitute the clinically actionable threshold warranting immediate oncological evaluation, whereas L1-L3 are typically managed conservatively or with short-interval follow-up [14,15]. Accordingly, L3 was included in the FNAC-negative group for binary diagnostic accuracy analysis; however, its intermediate ROM was analyzed separately in the ROM assessment and should not be interpreted as a true-negative category.

The mean of the three pathologists’ metrics is reported as the summary estimate; pooled statistics were not computed from an aggregate confusion matrix because the three pathologists assessed the same 427 specimens, and their assessments are therefore not independent observations. All proportions are presented with 95% confidence intervals (Wilson score method). Receiver operating characteristic (ROC) analysis and area under the ROC curve (AUC; with standard error by the Hanley-McNeil method) were computed per pathologist [16].

ROM was calculated as per the Sydney category, using Pathologist 1’s predefined reference classification as the proportion of cases confirmed malignant, with Wilson 95% CIs. Pathologist 1, the most senior pathologist among the three observers, was designated the a priori reference reader for ROM and TB 2 × 2 analyses by protocol prior to data unblinding, consistent with established practice in multi-reader validation studies to avoid post hoc selection of the best-performing observer.

TB correlation analysis was done by a complete 2 × 2 contingency table (L2 classification vs. ZN-positive TB status) to derive sensitivity, specificity, PPV, and NPV for TB detection by L2 classification, each with 95% CIs. For interobserver agreement, Cohen’s kappa (κ) was calculated for all three pairwise comparisons using pre-consensus classifications [17]. Agreement was interpreted as per Landis and Koch: κ < 0.20 = slight; 0.21-0.40 = fair; 0.41-0.60 = moderate; 0.61-0.80 = substantial; 0.81-1.00 = almost perfect [18].

## Results

Demographic characteristics and pathological findings

The age of patients ranged from 1 to 82 years (mean ± SD: 27.08 ± 15.54 years), consistent with the predominance of infectious and reactive lymphadenopathy in younger patients characteristic of TB-endemic regions. Of note, 68 patients (15.9%) were below 18 years of age; malignancy was confirmed in two of these pediatric cases (2.9%), both of which were lymphoproliferative disorders, consistent with the expected lower prevalence of malignancy in this age group. Demographic characteristics, nodal sites, presenting symptoms, cytopathological diagnoses, ZN staining results, and final diagnoses are presented in Table 1.

**Table 1:** Demographic characteristics, clinicopathological features, and diagnostic outcomes of the study cohort (n = 427) * Includes one case of ZN-staining-positive lepromatous leprosy classified as non-TB benign on histopathological examination. *Mycobacterium leprae* is weakly acid-fast and may produce ZN positivity; differentiation from mycobacterial TB requires histopathological integration. ZN: Ziehl-Neelsen, TB: tuberculosis, ALUS: atypical lymphoid cells of undetermined significance

Variable	Category	n (%)
Sex	Male	174 (40.7)
	Female	253 (59.3)
Nodal site	Cervical	357 (83.6)
	Axillary	48 (11.2)
	Inguinal	22 (5.2)
Presenting symptoms	Painless swelling	213 (49.9)
	Painful swelling	49 (11.5)
	Febrile episodes	76 (17.8)
	Significant weight loss	43 (10.1)
	Upper aerodigestive tract symptoms	31 (7.3)
	Shortness of breath	15 (3.5)
Cytopathological diagnosis	Reactive lymphadenitis	115 (26.9)
	Granulomatous lymphadenitis	120 (28.1)
	Necrotising lymphadenitis	119 (27.9)
	Granulomatous necrotizing lymphadenitis	25 (5.9)
	Lymphoproliferative disorder	6 (1.4)
	ALUS	8 (1.9)
	Inadequate specimen	5 (1.2)
	Metastatic carcinoma	29 (6.8)
ZN staining	Positive	74 (17.3)
	Negative	353 (82.7)
Final diagnosis	Non-tuberculous benign disease*	315 (73.8)
	TB	73 (17.1)
	Malignant disease	39 (9.1)

Sydney System category distribution per pathologist

All three pathologists independently reviewed 427 FNAC smears at separate time points and assigned Sydney System categories. Category distributions are presented in Table 2. The L2 category constituted the overwhelming majority of cases, with a mean of 384 (89.9%) across pathologists. One case initially classified as L2 by Pathologist 2 was reassigned to L5 following the predefined consensus review. For the primary kappa analysis, Pathologist 2’s original pre-consensus classification (L2) was retained, and the reclassified L5 designation was used only in the final diagnostic dataset.

**Table 2:** Distribution of Sydney System categories per pathologist (n = 427 specimens per pathologist) Kappa was computed on pre-consensus original classifications to preserve observer independence. ALUS: atypical lymphoid cells of undetermined significance

Sydney System category	Pathologist 1	Pathologist 2	Pathologist 3	Mean overall (%)
L1 - inadequate/non-diagnostic	5	2	1	0.62
L2 - benign/reactive	380	385	388	90.06
L3 - ALUS	8	8	6	1.72
L4 - suspicious for malignancy	5	4	5	1.09
L5 - malignant	29	28	27	6.56
Total	427	427	427	100

**Figure 1:**
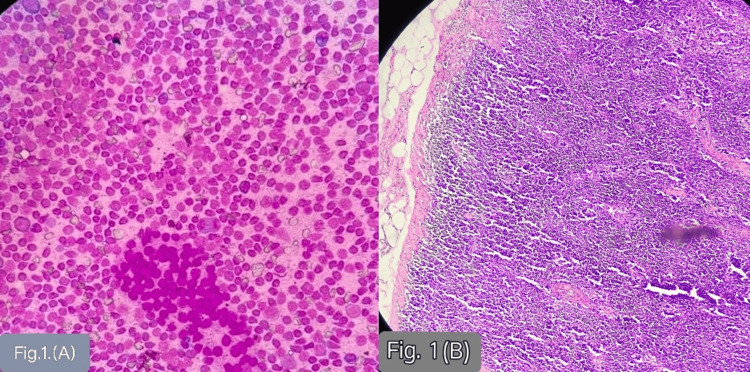
(A) FNA smear showing a polymorphous population of lymphoid cells comprising predominantly mature small lymphocytes admixed with immunoblasts. Categorized as L2: Benign. (Leishman stain; 10x magnification). (B) Histological section of the lymph node showing reactive follicular hyperplasia (hematoxylin and eosin stain; 10x magnification) FNA: fine-needle aspiration

Representative cytological and histopathological features of the L2 category are illustrated in Figure 1. The aspirate (Figure 1A) demonstrates cytomorphological features characteristic of reactive follicular hyperplasia and consistent with an L2 designation under the Sydney System. The background is clean without necrosis, atypical cells, or epithelioid granulomas. The corresponding histological section (Figure 1B) confirms reactive follicular hyperplasia (with appreciable lymph node architecture) at low power. This cytological-histopathological concordance illustrates the reliability of the Sydney System L2 category in identifying benign reactive lymphadenopathy and its strong negative predictive value (98.5% for Pathologist 1; 98.0% for Pathologists 2 and 3) for malignancy, supporting conservative clinical management in appropriately selected cases.

TB analysis

To evaluate the relationship between L2 classification and microbiologically confirmed TB, a complete 2 × 2 contingency table was constructed (Table 3) using Pathologist 1’s L2 classification (n = 380) as the analytical reference (Pathologist 1 being the pre-designated senior-reader reference, as specified in Methods). Of 74 ZN-staining-positive cases, 73 were confirmed TB, and 1 represented lepromatous leprosy (non-TB benign). From the complete matrix: TPᵀᴮ = 73 (L2 and ZN-positive TB), FPᵀᴮ = 307 (L2 and non-TB), TNᵀᴮ = 46 (non-L2 and non-TB), FNᵀᴮ = 1 (non-L2 and ZN-positive TB).

**Table 3:** Complete 2 × 2 analysis: Sydney system L2 classification versus ZN-confirmed TB (n = 427) * Specificity of L2 for TB = proportion of confirmed non-TB cases (n = 353; computed as 427 − 74 ZN-positive cases = 353) correctly classified as non-L2. ** PPV = proportion of L2-classified cases with microbiologically confirmed TB. *** NPV = proportion of non-L2-classified cases with confirmed non-TB disease. 95% CIs by Wilson score method. TPᵀᴮ: true positive for TB, FPᵀᴮ: false positive for TB (L2 but non-TB), TNᵀᴮ: true negative (non-L2 and non-TB), FNᵀᴮ: false negative for TB (non-L2 but ZN-positive TB) ZN: Ziehl-Neelsen, TB: tuberculosis, PPV: positive predictive value, NPV: negative predictive value, CI: confidence interval

Diagnostic parameter	Value	95% CI
L2 cases (Pathologist 1 reference)	380	-
ZN-confirmed TB in L2 (TPᵀᴮ)	73	-
Non-TB cases in L2 (FPᵀᴮ)	307	-
Non-L2 cases	47	-
ZN-confirmed non-TB among non-L2 (TNᵀᴮ)	46	-
ZN-confirmed TB among non-L2 (FNᵀᴮ)	1	-
Sensitivity of L2 for TB	98.6% (73/74)	92.7-99.8%
Specificity of L2 for TB*	13.0% (46/353)	9.9-16.9%
PPV of L2 for TB**	19.2% (73/380)	15.6-23.5%
NPV of non-L2 for non-TB***	97.9% (46/47)	88.9-99.6%

**Figure 2:**
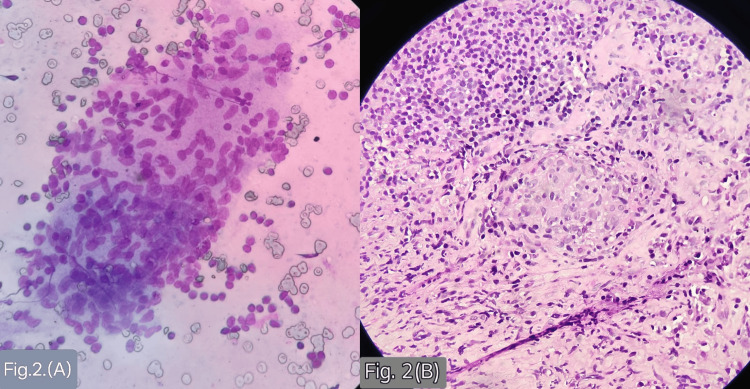
(A) FNA smear showing clusters of epithelioid cells forming granulomas with lymphoid cells in the background, consistent with granulomatous lymphadenitis. Categorized as L2: benign. (Leishman stain; 40x magnification). (B) Histological section of the lymph node showing epithelioid granuloma, confirming granulomatous inflammation. (Hematoxylin and eosin stain; 40x magnification) FNA: fine-needle aspiration

A morphologically distinct and clinically critical subset of L2 cases comprised granulomatous lymphadenitis, as illustrated in Figure 2. The cytomorphological features of the aspirate (Figure 2A) mandate ZN staining irrespective of clinical context; however, cytology alone cannot distinguish tuberculous from non-tuberculous granulomatous conditions. This case was confirmed as TB based on a positive ZN stain. The corresponding histological section (Figure 2B) supports the diagnosis by demonstrating epithelioid granulomatous inflammation with necrosis. We acknowledge that the representative photomicrograph does not display all classical histological features of tuberculous lymphadenitis within a single field.

These findings highlight a critical diagnostic discordance: although the Sydney System L2 category captures nearly all microbiologically confirmed TB cases (sensitivity 98.6%), its specificity for TB is only 13.0% and its PPV only 19.2%, meaning 80.8% of granulomatous cytological findings categorized as L2 are attributable to non-TB etiologies. This underscores the necessity of mandatory microbiological confirmation, ZN staining, mycobacterial culture, or nucleic acid amplification testing (e.g., GeneXpert MTB/RIF), before attributing an L2 classification to TB. Reliance solely on granulomatous cytomorphology risks overdiagnosis of TB and may delay identification of alternative conditions, including fungal infections, sarcoidosis, Kikuchi disease, and malignancy.

Diagnostic accuracy for malignancy

**Figure 3:**
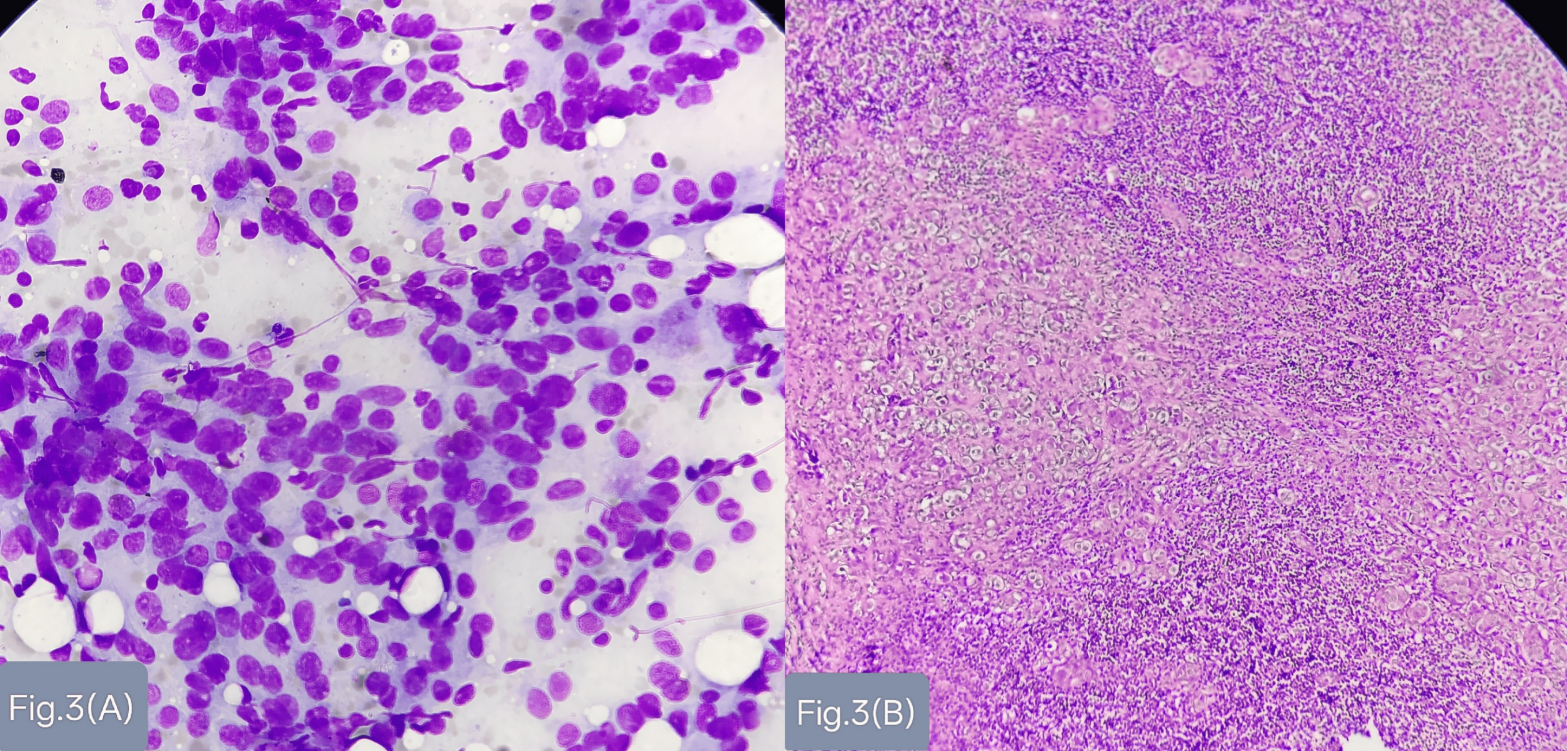
(A) FNA smear showing loosely cohesive clusters and dispersed pleomorphic tumor cells with a high nuclear-to-cytoplasmic ratio, nuclear overlapping, and prominent nucleoli. Categorized as L5: malignant. (May-Grunwald-Giemsa stain; 40x magnification). (B) Histological section of the lymph node showing effacement of normal nodal architecture by sheets of malignant cells, confirming the cytological diagnosis of malignancy. (Hematoxylin and eosin stain; 10x magnification) FNA: fine-needle aspiration

The reference standard for malignancy was histopathological examination. Cases assigned L4 or L5 were designated FNAC-positive; L1-L3 were FNAC-negative. The L5 is illustrated by the representative case in Figure 3. The aspirate (Figure 3A) is highly cellular, demonstrating cytomorphological findings that collectively fulfill the criteria for an unequivocal L5 designation under the Sydney System. The corresponding histological section (Figure 3B), examined at low power to demonstrate the extent of architectural disruption, shows complete effacement of normal nodal architecture by sheets of malignant cells, confirming the FNAC diagnosis.

Among the 388 (90.8%) confirmed non-malignant cases, histopathological biopsy results were available for 214 (55.2%); the remaining 174 (44.8%) were confirmed using the composite criteria described in the Methods section. Diagnostic performance metrics with 95% confidence intervals are presented in Table 4.

**Table 4:** Diagnostic accuracy parameters for malignancy detection (n = 427; confirmed malignant = 39; confirmed non-malignant = 388). All percentages in parentheses represent 95% Wilson confidence intervals Summary column = mean ± SD of three pathologists. 95% CIs computed by the Wilson score method. n= total number of cases, sensitivity = TP/(TP+FN), specificity = TN/(TN+FP), PPV = TP/(TP+FP), NPV = TN/(TN+FN), accuracy = (TP+TN)/n TP: true positive, FP: false positive, TN: true negative, FN: false negative, PPV: positive predictive value, NPV: negative predictive value, SD: standard deviation

Parameter	Pathologist 1	Pathologist 2	Pathologist 3	Mean across pathologists
TP	33	31	31	31.7
FP	1	1	1	1
TN	387	387	387	387
FN	6	8	8	7.3
Sensitivity	84.6% (70.3-92.8%)	79.5% (64.5-89.2%)	79.5% (64.5-89.2%)	81.2% (SD 2.4%)
Specificity	99.7% (98.6-100.0%)	99.7% (98.6-100.0%)	99.7% (98.6-100.0%)	99.7% (SD 0.0%)
PPV	97.1% (85.1-99.5%)	96.9% (84.3-99.4%)	96.9% (84.3-99.4%)	96.9% (SD 0.1%)
NPV	98.5% (96.7-99.3%)	98.0% (96.1-99.0%)	98.0% (96.1-99.0%)	98.1% (SD 0.2%)
Overall accuracy	98.4% (96.7-99.2%)	97.9% (96.0-98.9%)	97.9% (96.0-98.9%)	98.0% (SD 0.2%)

The mean of the three pathologists’ estimates is reported as the summary; a single pooled confusion matrix aggregating three assessments of the same 427 specimens was not computed because it would violate the independence assumption and inflate the effective sample size. The ROC statistics are presented in Table 5.

**Table 5:** AUC for malignancy discrimination per pathologist (Hanley-McNeil SE; 95% CI by the normal approximation) Statistical significance was defined as p < 0.05. All AUC values were significantly different from the null hypothesis of no discrimination (AUC = 0.5); significant p-values are denoted with *. AUC: area under the receiver operating characteristic curve, SE: standard error of the AUC estimate, CI: confidence interval

Pathologist	AUC (mean ± SE)	p-value
Pathologist 1	0.974 ± 0.018 (95% CI: 93.9-100.0%)	<0.001*
Pathologist 2	0.935 ± 0.028 (95% CI: 88.1-98.9%)	<0.001*
Pathologist 3	0.935 ± 0.028 (95% CI: 88.1-98.9%)	<0.001*

ROM per Sydney category

ROM was calculated from Pathologist 1’s predefined reference classification (Table 6). A stepwise increase is observed from L2 to L5, consistent with the Sydney System's hierarchical risk-stratification intent. The higher ROM in L1 than in L3 is consistent with the published literature, which reports that inadequate specimens are disproportionately obtained from clinically suspicious lesions. However, caution is warranted in interpreting ROM estimates for L1, L3, and L4 categories, which contain small numbers of cases (n = 5, 8, and 5, respectively); the 95% CIs for these categories are correspondingly wide, and these point estimates should be interpreted as preliminary in this cohort.

**Table 6:** ROM per Sydney system category with 95% Wilson CIs (n_total = 427, n_malignant = 39) ROM: confirmed malignant cases in category/total cases in category × 100, ALUS: atypical lymphoid cells of undetermined significance, CIs: confidence intervals

Category	Total cases (n)	Confirmed malignant (n)	ROM (%) (95% CI)
L1 – inadequate/non-diagnostic	5	1	20.0% (3.6-62.4%)
L2 – benign/reactive	380	4	1.1% (0.4-2.7%)
L3 – ALUS	8	1	12.5% (2.2-47.1%)
L4 – suspicious for malignancy	5	4	80.0% (37.6-96.4%)
L5 – Malignant	29	29	100.0% (88.3-100.0%)
Total	427	39	-

Interobserver agreement

Cohen’s kappa coefficients computed from pre-consensus independent classifications are presented in Table 7. Values of κ = 0.79-0.87 indicate substantial to almost perfect agreement (Landis and Koch classification), confirming robust reproducibility of the Sydney System across independent reviewers. Minor variability was concentrated at the L2/L3 boundary, reflecting the recognized challenge of distinguishing mild reactive atypia from true atypical lymphoid cells of undetermined significance.

**Table 7:** Interobserver agreement (Cohen’s kappa) among three pathologists applying the Sydney system (pre-consensus classifications) Agreement strength (Landis and Koch): κ 0.61-0.80 = substantial, κ 0.81-1.00 = almost perfect

Pathologist pair	κ value	Agreement strength (Landis and Koch)
Pathologist 1 vs. Pathologist 2	0.87	Almost perfect
Pathologist 1 vs. Pathologist 3	0.82	Almost perfect
Pathologist 2 vs. Pathologist 3	0.79	Substantial

## Discussion

The present study validates the Sydney System for reporting lymph node cytopathology in a TB-endemic tertiary care setting in Eastern India. It demonstrates its strong diagnostic utility, reproducibility, and clinical applicability. Per-pathologist sensitivity ranged from 79.5% to 84.6%; specificity was 99.7% across all three observers; PPV was 96.9-97.1%; and NPV was 98.0-98.5%. These findings are consistent with previously published validation studies and reinforce the value of the Sydney System as a standardized framework for reporting lymph node FNAC [7-10].

FNAC remains the preferred first-line investigation for peripheral lymphadenopathy because it is rapid, minimally invasive, cost-effective, and well-suited to resource-constrained settings. In countries such as India, where TB and reactive inflammatory lesions constitute a substantial proportion of lymph node pathology, FNAC plays an especially important role in reducing unnecessary surgical biopsies. [2,3] Prior to the Sydney System, reporting terminology lacked uniformity, resulting in inconsistent interpretation and variable clinical management. The Sydney System addressed this by introducing a structured five-tier classification linked to ROM and suggested management pathways [4].

A progressive increase in ROM from lower to higher Sydney categories was observed: 1.1% for L2, 12.5% for L3, 80.0% for L4, and 100.0% for L5. Caution is warranted when interpreting ROM estimates for L1 (n = 5), L3 (n = 8), and L4 (n = 5), as small sample sizes yield wide 95% confidence intervals; these estimates should be regarded as preliminary for this cohort. Notwithstanding, the directional stepwise increase is consistent with findings reported by Gupta et al. and Alqaidy et al. [14,15]. The near-perfect ROM in L5 (100.0%) confirms that cytological malignant categorization is highly predictive of true malignancy and warrants prompt histopathological confirmation and oncological management.

The high specificity of 99.7% is clinically significant because it minimizes false-positive malignant diagnoses and unnecessary invasive procedures. The comparatively lower sensitivity (range 79.5-84.6%) may reflect cytomorphological overlap between reactive lymphoid hyperplasia and low-grade lymphoid neoplasms, particularly when atypia is scant, a recognized diagnostic pitfall in lymph node cytology [19]. Similar sensitivity limitations have been reported in previous Sydney System validation studies [7,14]. Ancillary techniques (flow cytometry, immunocytochemistry, and cell block preparation) may improve diagnostic confidence in atypical and suspicious categories and warrant systematic incorporation in future protocols [2].

One of the most important observations is the predominance of granulomatous and reactive lesions within L2, with a mean of 384 (90.9%) out of 427 aspirates, reflecting the high prevalence of these conditions in TB-endemic populations. The complete TB analysis reveals a critical diagnostic discordance: while L2 classification captures nearly all microbiologically confirmed TB cases (sensitivity 98.6%), its specificity for TB is only 13.0%, and its PPV is only 19.2%. This means that more than four in five granulomatous aspirates categorized as L2 are attributable to non-TB etiologies, including fungal infections, sarcoidosis, Kikuchi disease, toxoplasmosis, and certain malignancies. [12,20] These findings strongly support the Index-TB Guidelines' emphasis on microbiological confirmation using ZN staining, culture, or nucleic acid amplification (GeneXpert MTB/RIF) in extrapulmonary TB before initiating anti-TB therapy. [21] Reliance solely on granulomatous morphology may lead to overdiagnosis of TB and delay diagnosis of treatable alternative conditions.

Interobserver agreement was substantial to almost perfect (κ = 0.79-0.87), consistent with earlier Sydney System validation studies [8,14,15] and confirming reproducibility across independent pathologists. Minor variability was predominantly observed at the L2/L3 boundary, which is expected given the subjective nature of mild lymphoid atypia on cytology smears. [9] Kappa statistics were interpreted according to the Landis and Koch criteria. [18] AUC values ranged from 0.935 to 0.974 across the three pathologists, further strengthening the discriminative validity of the Sydney System. [16].

This study has several strengths. It is among the few multicentric validations of the Sydney System from Eastern India, a region with a high burden of TB and reactive lymphadenopathy. [11] Three independent pathologists from two separate institutions reviewed all slides at separate time points, substantially minimizing observer and recall bias. Critically, a complete TB 2 × 2 matrix-based analysis, including specificity, underreported in several published validations, was performed, providing a more complete picture of the system's TB-diagnostic limitations. Pediatric and adult patients were included, reflecting the real-world practice at these centers.

Several limitations require acknowledgment. The retrospective observational design precluded the systematic application of molecular tests (e.g., GeneXpert MTB/RIF) to all granulomatous aspirates; histopathological confirmation was unavailable for all benign cases. [13] The L3, L4, and L1 category ROM estimates are based on small case numbers and should be replicated in larger cohorts. Ancillary investigations (flow cytometry, immunocytochemistry) were not systematically performed in atypical cases. [2] Future multicentric studies incorporating these techniques and molecular diagnostics may further refine the diagnostic performance of the Sydney System in TB-endemic settings.

Overall, these findings support adoption of the Sydney System as the standard reporting framework for lymph node FNAC in tertiary care centers, particularly in resource-constrained and TB-endemic settings [4,5].

## Conclusions

This multicentric validation study from a TB-endemic region of Eastern India demonstrates that the Sydney System for reporting lymph node cytopathology is a reliable, reproducible, and clinically actionable framework for lymph node FNAC, showing high diagnostic accuracy for malignancy and excellent interobserver agreement. The progressive risk-of-malignancy gradient across categories supports clinically appropriate triage, from conservative management of lower-risk lesions to expedited evaluation of suspicious and malignant cases. Importantly, granulomatous cytomorphology alone was insufficient for diagnosing TB, underscoring the need for mandatory microbiological confirmation before attributing L2 granulomatous lesions to tuberculosis in endemic settings. Integration of ancillary tests, including ZN staining, nucleic acid amplification testing, flow cytometry, and immunocytochemistry, may further enhance diagnostic performance. These findings support adoption of the Sydney System as a standard reporting framework for lymph node FNAC in resource-limited, TB-endemic settings.
